# Case Report: Lethal mitochondrial cardiomyopathy linked to a compound heterozygous variant of *PARS2*

**DOI:** 10.3389/fcvm.2024.1446055

**Published:** 2024-08-26

**Authors:** Siyuan Jing, Qiuyan Yao, Mei Wu, Yifei Li

**Affiliations:** ^1^ Key Laboratory of Birth Defects and Related Diseases of Women and Children of MOE, Department of Pediatrics, West China Second University Hospital, Sichuan University, Chengdu, Sichuan, China; ^2^ Department of Nursing, West China Second University Hospital, Sichuan University, Chengdu, China

**Keywords:** *PARS2*, WES, mitochondrial disorder, dilated cardiomyopathy, heart failure

## Abstract

**Introduction:**

Variants in the *PARS2* gene have been previously associated with developmental and epileptic encephalopathy. *PARS2* deficiency was characterized as a neurodevelopmental and neurodegenerative disorder with early-onset seizures and global developmental delay. Herein, we reported the first case with severe heart failure due to lethal mitochondrial cardiomyopathy with *PARS2* compound heterozygous variants.

**Case presentation:**

This patient demonstrated fatigue, chest tightness, and shortness of breath. An acute major illness had been identified at the initial evaluation, which was characterized by severe diaphoresis, dizziness, and fatigue. Blood–urine tandem mass spectrometry found multiple disorders in acid metabolism, characterized as increased homovanillic acid (130.39 mmol/L) and 2-hydroxyisovaleric acid (1.70 mmol/L), which are associated with myocardial injuries. Therefore, an inherited metabolic disorder was suspected and whole-exome sequencing was performed, revealing a novel compound heterozygous variant of c.953C>T and c.283G>A on *PARS2*. Echocardiography confirmed the findings from the MRI, which presented an increased left ventricular diameter at the end of the diastolic stage. The molecular structure of SYPM was established as AF-Q7L3T8-F1, and the identified mutant sites were located in the proline-tRNA ligase domain. However, the patient died due to severe heart failure.

**Conclusion:**

This is the first case to reveal a novel compound heterozygous variant of *PARS2*-induced lethal cardiomyopathy with unreversed heart failure. Thus, this report enhances our understanding of mitochondrial tRNA function in maintaining heart function.

## Introduction

Inherited mitochondrial disorders often lead to severe systemic dysfunction, including developmental delays, neurological impairments, cardiomyopathy, and muscle weakness (1). These conditions can be fatal before adulthood, posing significant challenges for medical treatment and familial care (2). Mitochondria are encoded both by nuclear and mitochondria genomes (3). While variants in mitochondrial DNA (mtDNA) can cause a range of mitochondrial diseases, they are maternally inherited, and the disease's severity is determined by the proportion of affected mitochondria. Moreover, increasing numbers of variants had been identified to be associated with mitochondrial disorders due to the development of next-generation sequencing (NGS), including electron transport chain components, mitochondrial member formation proteins, mtDNA transcription activator proteins, mtDNA translation proteins, and mitochondrial aminoacyl-tRNA synthetases (aaRSs). Among them, a deficiency in aaRSs has been considered as the fundamental genes for mitochondrial protein biosynthesis and oxidative phosphorylation (4). For mitochondrial disorders, neurological manifestation and myocardial injuries commonly affect patients of a young age, including recurrent seizures and sustainable myocardial injuries. Mitochondrial cardiomyopathy is a myocardial disorder characterized by abnormal myocardial structure and/or function due to genetic defects that impair the mitochondrial respiratory chain, absence of coronary artery disease, hypertension, valvular disease, or congenital heart disease (5). Moreover, the clinical prognosis of mitochondrial cardiomyopathy is critically poor. Early diagnosis and promotion of treatment would benefit the patients. Thus, expanding the knowledge of mitochondrial disorder–related diseases provide more information for managing such cases. It is estimated that mitochondrial cardiomyopathy accounts for approximately 20%–40% of children with mitochondrial diseases. Zhang et al. used a *Tfam* conditional knock out mice transgenetic model to demonstrate that the molecular mechanism of mitochondrial dysfunction induced cardiomyopathy by activating DNA damaging associated cell cycle arrest (6). They also established a DOX-induced *Tfam* knock in induced pluripotent stem cell (iPSC) model to reveal the mitochondria in ectoderm and mesoderm formation, underlining the developmental mechanisms in mitochondria-associated encephalopathy and cardiomyopathy (7).

Among the 19 genes that have been identified as belonging to aaRSs, *PARS2* had been reported in 10 studies, which drew an identical association between *PARS2* variants and developmental and epileptic encephalopathy (DEE) (8). According to previous reports, *PARS2* deficiency was characterized as a neurodevelopmental and neurodegenerative disorder with early-onset seizures and global developmental delay (9). Half of the affected patients died around 10 years of age (10). However, some of them survived to adulthood. Until now, there have been 12 patients who had detailed documented medical phenotype and therapeutic information. However, most of them presented with severe recurrent seizures and aggressive progressed encephalopathy. A few patients have been reported with reduced heart function, and most of them presented with a slight reduction in left ventricular ejection fraction (LVEF) or even normal contractile function and structure. Accordingly, the issue of cardiomyopathy has not been raised by physicians and researchers, and no case has been described in which a patient died due to heart failure.

Herein, we reported the first case with severe heart failure due to lethal mitochondrial cardiomyopathy with a compound heterozygous variant on *PARS2* [c.283G>A, p.V95I (paternally inherited) and c.953C>T, p.S318l (maternally inherited)]. This proband presented epilepsy as the early-onset manifestation, while no worsening neurological symptom had been identified since 5 years of age. However, dilated cardiomyopathy (DCM) with aggressive decreased heart function was recorded, and the proband finally died due to sudden cardiac arrest. This case highlights the link between impaired mitochondrial bioenergetics and cardiomyopathy in addition to neurological diseases and demonstrates the importance of early assessments in cardiac function for mitochondrial disorders and the requirements of early intervention to maintain heart function.

## Case presentation

### Medical history and physical examination

This study was approved by the Ethics Committee of West China Second Hospital of Sichuan University (2021-069). We obtained written informed consent from the parents of the participant who was under the age of 16 years to participate in this research before performing exome sequencing and for the inclusion of the patient's clinical and imaging details in subsequent publications.

The proband was a 9-year-old male, who was admitted to our hospital with a history of decreased activity tolerance for over a year and presented with severe fatigue and shortness of breath for 6 h. Moreover, the proband experienced sudden cardiac arrest 30 min before attending the hospital, after which he recovered consciousness in 2 min. The patient was transferred to the emergency department. The patient had usually complained with shortness of breath and chest tightness 1 year before. In addition, a significant reduction in activity tolerance had been observed. Then, such clinical manifestations appeared to rapidly worsen. Six months before the most recent hospital admission, the proband found it challenging to climb stairs, and he was classified as New York Heart Association (NYHA) class III. 2162;. At that time, the proband consulted cardiac physicians in our hospital and DCM had been suspected with reduced heart contractile function and enlarged left atrium and ventricle. Thus, Entresto, digoxin, spironolactone, and furosemide were provided to this patient, and he showed slight improvements in activity tolerance and shortness of breath. Recently, the patient presented with a respiratory infection with severe cough and fever. Oral antibiotics had been taken with other medication to relieve cough and fever. Unfortunately, heart function seemed to decreased rapidly with noticed arrhythmia, presenting with worsening fatigue and shortness of breath. Finally, cardiac arrest was recorded.

The patient had a history of severe neurological developmental impairment since 2 years old, characterized by recurrent seizures diagnosed as epilepsy. Sodium valproate was administered for 5 years, with no seizures observed in the last 4 years, indicating stable neurological function.

At admission, the heart rate of the patient was 130 beats per min. The blood pressure was measured at 75/45 mmHg. The respiratory rate was 45 breaths per min. There were no surface wounds in the chest and other areas, indicating the absence of accidental injuries. Physical examination revealed pitting edema in the lower extremities, moderate jaundice, yellowing of the conjunctiva, pharyngeal congestion, and rough respiratory sounds with wet rales. The heart sounds were weak and irregular without murmurs, and the liver was palpable 7 cm below the rib cage. Neurological examination did not show any abnormalities. The extremities were warm with a capillary refill time of 3 s. Muscle strength and tension of four extremities were normal, and no pathological or meningeal irritation signs were observed.

In addition, the parents of the proband failed to provide any positive family history of hypertension, coronary artery diseases, cardiac arrest, diabetes, or obesity. No hereditary diseases such as cardiomyopathy and metabolic disorders were found in the family. Furthermore, his parents did not show any neurological or cardiovascular symptoms.

### Imaging and laboratory examinations

Rapid blood gas analysis identified an unexplained acidemia (pH 7.046) with hyperlactatemia (lactate 15.18 mol/L). Laboratory assessment revealed a significantly elevated B-type natriuretic peptide level (>5,000 pg/ml, negative value [n.v.] <100 pg/ml) and mildly increased cTnI (0.176 µg/L, n.v. <0.058 µg/L). Hepatic and renal injuries were also observed. Blood–urine tandem mass spectrometry found multiple disorders in acid metabolism, characterized as increased homovanillic acid (130.39 mmol/L, n.v. <42.35 mmol/L) and 2-hydroxyisovaleric acid (1.70 mmol/L, n.v. <0.50 mmol/L), which are associated with myocardial injuries.

**Figure 1:**
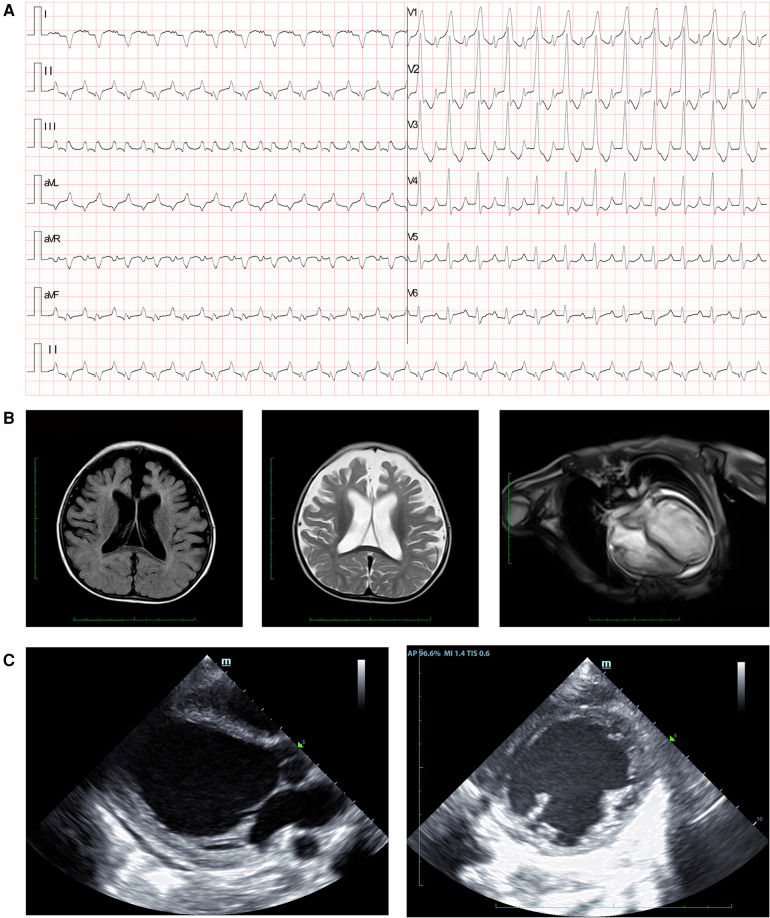
Clinical manifestation of the proband. **(A)** Electrocardiogram findings showed atrial tachycardia, completed right branch bundle block, and right ventricular hypertrophy. **(B)** Cranial MRI demonstrated brain atrophy in the proband. Myocardial MRI showed general enlarged cardiac ventricles, reduced contractile function, and delayed myocardial enhancement. **(C)** Echocardiography revealed increased left ventricular diameter at the end of the diastolic stage.

The patient's history of illness, physical examination, and laboratory tests strongly suggested severe heart failure, characterized by atrial tachycardia, complete right branch bundle block, and right ventricular hypertrophy (Figure 1A). Cardiac MRI assessments revealed mild brain atrophy (Figure 1B), general enlarged cardiac ventricles, mainly affected left ventricle (LV) (left ventricular internal diameter end diastole = 43.6 mm, right ventricular internal diameter end diastole = 38.9 mm), reduced contractile function (LVEF = 47.6%, stroke volume = 49.8 ml, end diastolic volume = 105.7 ml, end systolic volume = 54.9 ml), and delayed myocardial enhancement in LV. Echocardiography demonstrated global heart enlargement (LV = 47 mm, right ventricle = 18 mm), decreased LVEF (10%, biplane Simpson's method), and mild multi-valve insufficiency (mitral valves and tricuspid valves) (Figure 1C). Moreover, echocardiography scans had been conducted on the proband's parents to assess their clinical characteristics, and the results were negative.

Subsequently, systematic screening was performed to identify the potential cause of myocarditis (infective or autoimmune-related). The autoimmune antibody and rheumatic analyses were negative in this patient, while the erythrocyte sedimentation rate remained at a normal level. Potential tuberculosis infection was eliminated by T-Spots and Xpert tests. Furthermore, the test results for viral antigens and qPCR detecting viral DNA tests were negative, ruling out viral infection. Bacterial cultures of blood and sputum also failed to detect associated infectious pathogens. Therefore, autoimmune- or infection-related myocarditis had been excluded and inherited or genetic variant-cardiomyopathy was suspected.

### Molecular results

The genetic tests were performed with samples from the proband and their parents. The peripheral blood samples were obtained from them in an ethylenediaminetetraacetic acid anticoagulant blood sample tube and then stored at 4 °C for less than 6 h. DNA was extracted using the Blood Genome Column Medium Extraction Kit (Tiangen Biotech, Beijing, China) according to the manufacturer's instructions. Whole-exome sequencing (WES) was performed using the NovaSeq 6000 platform (Illumina, San Diego, CA, USA). Variant annotation was performed in accordance with the database-sourced minor allele frequencies (MAFs) and the practical guidelines of pathogenicity according to the American College of Medical Genetics. The annotation of MAFs was performed using the 1000 Genomes, ExAC, and Chigene inhouse MAF databases. MutationTaster and SIFT with R software were used to predict the pathogenicity.

**Figure 2:**
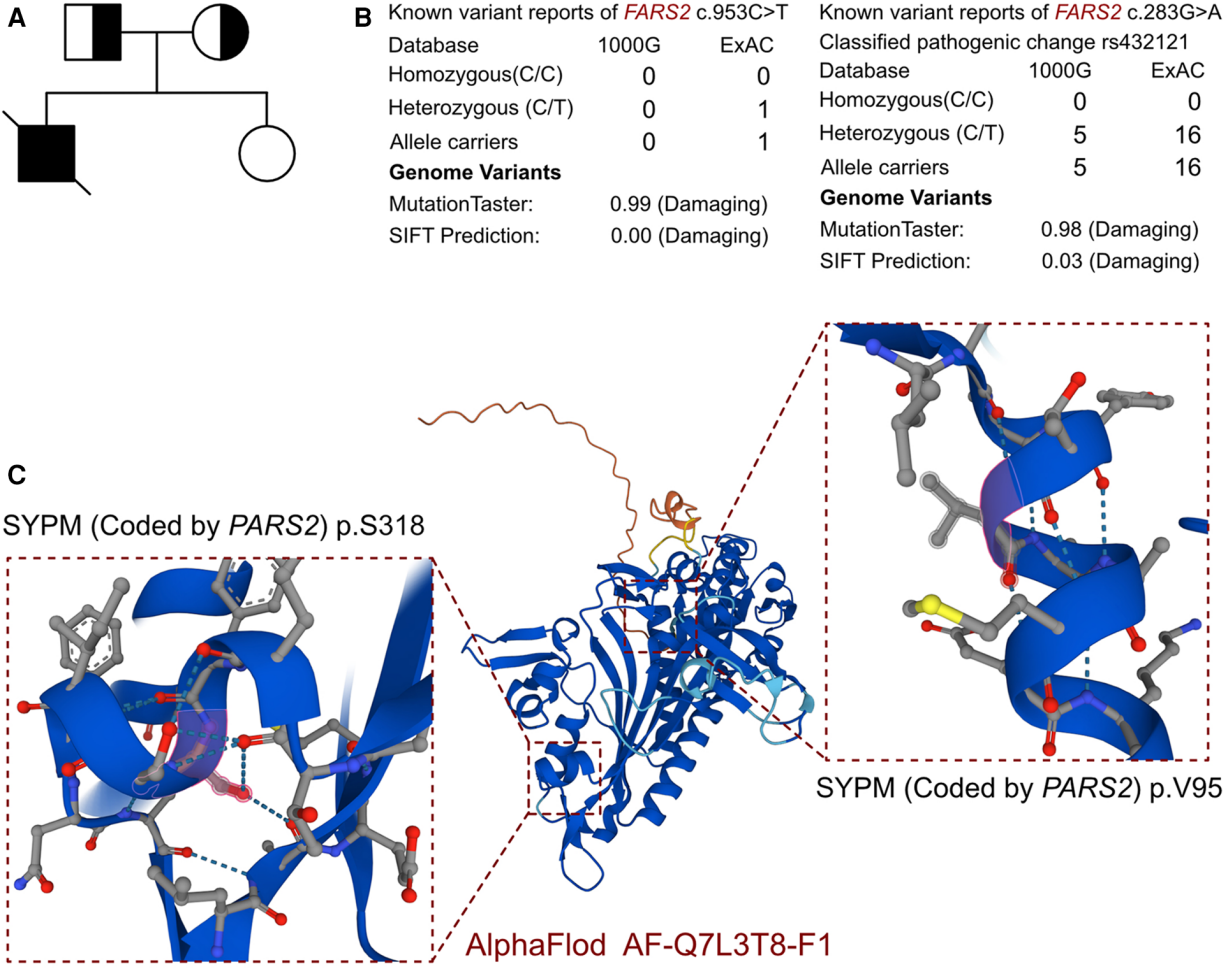
The molecular features of *PARS2*. **(A)** The proband exhibited a *de novo* compound heterozygous variant of *PARS*. **(B)** The variants of *PARS*, c.283G>A and c.953C>T, have been reported in 1000 Genomes and ExAC and were predicted to be protein damaging by MutationTaster. **(C)** The protein structure of SYPM was built, named AF-Q7L3T8-F1.

The clinical presentation and laboratory results strongly suggested a genetic basis for the disease. Whole-exome sequencing revealed a novel compound heterozygous variant in the *PARS2* gene (c.283G>A, p.V95I; c.953C>T, p.S318l), which we propose as a novel finding associated with cardiomyopathy (Figure 2A). The variant site of c.283G>A has been reported in several individuals and is considered a pathogenic site (rs432121), while there are few reports on the c.953C>T site (Figure 1B). Although there were several records that could be found from the 1000 Genomes and ExAC databases of the two identified variants (Figure 2B), this was the first study to report a compound heterozygous variant (c.283G>A and c.953C>T) associated with *PARS2*-induced cardiomyopathy. Therefore, we considered this compound variant as a novel finding in cardiomyopathy patients.

In addition, other potential pathogenic variants were evaluated. The search strategy contained three steps. The first was to evaluate the pathogenicity among all the variants reported as pathogenic and likely pathogenic. The second step was to review all the variants reported as uncertain which were expressed in cardiomyocytes. Finally, we assessed all the variants located in all the genes related to cardiomyopathy based on previous reports. In this case, beyond the *PARS2* gene, no other potential variant had been identified in the proband. There were no other cardiovascular-related variants between this proband and his parents. As there was no available full-length protein crystal structure for SYPM (encoded by *PARS2*), which had been analyzed by X-ray and cryogenic electron microscopy, AlphaFold protein structure software (https://alphafold.ebi.ac.uk/) was used to predict the crystal structure of the protein. The protein structure of SYPM was built and named AF-Q7L3T8-F1, and the identified mutant sites located in the proline-tRNA ligase domain. The mutation site of c.283G>A was located in the dimer interface of the ligase core domain, and it induced amino acid sequence change, the protein structure was affected, and the slice site changed, while the mutation site of c.953C>T only induced amino acid sequence changes. According to MutationTaster analysis, both variants are considered to be disease-causing mutations, and the probability values were 0.98 and 0.99, respectively. The SIFT scores predicted both of the variants to be protein damaging (0.00).

### Final diagnosis and treatment

After a series of examinations and molecular tests, the final diagnosis of the patient was aggressive progressed heart failure due to *PARS2*-associated cardiomyopathy. As the patient had severe heart failure, the primary therapeutic objective was to alleviate and improve the heart failure symptoms. During hospitalization, the patient was treated with a combination of epinephrine, norepinephrine, milrinone, dobutamine, spironolactone, and furosemide to manage the heart failure. Aspirin was administered for anticoagulation purposes, while prednisone acetate and methotrexate were prescribed for anti-inflammatory effects. Sacubitril valsartan sodium (100 mg twice a day) and empagliflozin (10 mg daily) were used to enhance and maintain cardiac contractile function. As the patient had not demonstrated epilepsy in recent years, antiepileptic drugs were not provided at this stage. During his hospital stay, severe acidosis was observed (lowest pH value was 6.946 and highest lactate was 16.65 mmol/L), and worsening heart function induced lower blood pressure. On the 4th day of hospitalization, the patient died due to heart failure.

## Discussion

In the past 25 years, advancements in genetic research have elucidated the molecular foundations of mitochondrial cardiomyopathy, identifying over a thousand mutations across various genes involved in mitochondrial function (11). This case report is the first to report a compound heterozygous variant in *PARS2* causing lethal cardiomyopathy, expanding the known spectrum of *PARS2*-related disorders. Genetic causes of primary mitochondrial disease may be subdivided into defects in oxidative phosphorylation subunits and complex assembly, disorders of mtDNA maintenance, defects in mitochondrial gene expression (such as mitochondrial aminoacyl-tRNA synthetases), deficiencies in cofactor biosynthesis and transport, defects in mitochondrial solute and protein import, disorders of mitochondrial lipid membranes and organellar dynamics (fission/fusion), and disorders of mitochondrial quality control (12). Despite the complex clinical phenotypes of mitochondrial diseases, leukoencephalopathy is now recognized to be a feature of many of the mitochondrial translation disorders, particularly aminoacyl-tRNA synthetase deficiencies, while insufficient adenosine triphosphate (ATP) production is more associated with diseases such as Parkinson's disease (13, 14). Meanwhile, these defects underscore the variety of molecules and pathways implicated in hypertrophic, dilated, restrictive, and arrhythmogenic cardiomyopathies (15). Consequently, screening for cardiomyopathy in patients with known or suspected mitochondrial disease is a standard part of managing the disease in both children and adults. The severity of mitochondrial cardiomyopathies varies from asymptomatic conditions to severe manifestations such as heart failure, arrhythmias, and sudden cardiac death (11). These symptoms are often accompanied by other signs of multi-organ involvement typical of mitochondrial diseases. However, mitochondrial cardiomyopathy can manifest without known mitochondrial disease, potentially being the first or sole clinical manifestation. Mutations in several mitochondrial tRNA genes (e.g., MTTK causing MERRF syndrome and MTTL1 causing MELAS syndrome) have been associated with multi-organ mitochondrial diseases or isolated cardiomyopathies (16–19). Cardiomyopathies associated with pathogenic mutations encoding mitochondrial tRNA genes are usually hypertrophic, but can also exist as dilated or histiocytoid cardiomyopathy. Hypertrophic cardiomyopathy (HCM) has been reported with mutations in the mitochondrial 16S rRNA gene (*MTRNR2*) (18, 19) and restrictive cardiomyopathy with the m.1555A>G mutation in the mitochondrial 12S rRNA gene (*MTRNR1*), typically linked with aminoglycoside-induced hearing loss (20, 21). Combination phenotypes involving cardiomyopathy and encephalopathy are common in mitochondrial diseases, affecting organs with the highest metabolic demands, such as the nervous system and the muscular system. Common symptoms include encephalopathy, myopathy, hearing loss, and diabetes.

Humans have 37 aaRSs, of which 18 are only active in the cytoplasm, 17 exclusively in mitochondria, and 2 in both compartments. Due to the importance of aaRSs, their mutations often cause a wide range of tissues or organs to be affected, with the nervous system being the most common and most severely affected organ. However, it was also clinically found that patients carrying *AARS2*, *KARS*, and *PARS2* mutations showed the phenotype of hypertrophic cardiomyopathy (22, 23). Moreover, one research study on FARS2 found that seven patients carrying FARS2 gene mutations manifested as hypertrophic cardiomyopathy, and experimentally proved that the gene mutation affected mitochondrial homeostasis, ultimately leading to myocardial hypertrophy and heart failure (24). The *PARS2* gene encodes mitochondrial prolyl-tRNA synthetase 2, a dimer composed of two identical protein monomers (9, 25). Each monomer contains a ligase core domain and an anticodon-binding domain. There were three active motifs in the ligase core domain: motif 1 is involved in the dimer interface; motif 2 couples ATP, amino acids, and tRNA binding; and motif 3 binds ATP. The anticodon-binding domain recognizes the anticodon loop of the cognate tRNA. Pathogenic variants in these functional regions can cause DEE75. The DEE75 phenotype is highly variable with systemic features. Neurological symptoms include profound developmental delay (100%), seizures (79%), cerebral atrophy (86%), and impaired intellectual development (100%) (26). Some patients exhibited the involvement of other organs, including the liver, kidneys, eyes, muscles, and cardiac system. The precise correlation between genotype and phenotype remains unknown. Since a complete loss of function in each mitochondrial aaRS is incompatible with life, the severity of the phenotype may be associated with the residual activities of the mutated synthetases. Thus far, no patients with biallelic loss-of-function variants have been reported (26, 27).

We have systematically reviewed all the reported cases with compound *PARS2* genetic variants, and 14 patients were retrieved (8–10, 25, 26, 28–30). All the identified cases presented early-onset poor growth from dozens of months to 2.5 years. Five patients died due to *PARS2*-associated mitochondrial disorder and the youngest age was 4 months, while the oldest was 21 years. The remaining patients lived, and three patients survived past 20 years old. Global developmental delay was the dominant phenotype associated with *PARS2* variants, while recurrent seizure attacks were observed among most of the patients. We then summarized the cardiac involvements among the patients, and only four patients were found to have abnormal myocardial structure, mainly indicating HCM. Only one case with the biallelic variants of c.283G>A and c.1091C>G who died at 21 years of age demonstrated a reduction of LVEF at 20% (9). There was no available case to link the *PARS2* variants with severe mitochondrial cardiomyopathy leading to lethal heart failure. Thus, it is essential to evaluate cardiac function and fibrosis in *PARS2*-variant-associated mitochondrial disorder and the promotion of therapeutic interventions for cardiac protection should be considered for such patients.

Mitochondrial cytopathies are linked with reduced aerobic energy transduction (decreased ATP synthesis), increased oxidative stress, apoptosis, and necrosis. The elevated levels of homovanillic acid and 2-hydroxyisovaleric acid in this patient suggest that *PARS2* may lead to abnormal metabolism of amino acids and catecholamines by affecting mitochondrial homeostasis. Furthermore, the results of relevant studies show that its level can be used to predict and evaluate the severity of disease (31, 32). In theory, certain combinations of nutraceuticals can overcome such deficiencies in mitochondrial function, potentially offering an alternative energy source. In a previous randomized placebo-controlled trial conducted with patients with mitochondrial cytopathies, it was reported that alpha-lipoic acid and coenzyme Q10 decreased lactate levels and oxidative stress markers. Recently, researchers demonstrated that the novel medications empagliflozin and sacubitril valsartan sodium had the potential to improve mitochondrial function and maintain cardiac function (33, 34).

However, there is a limitation in the research. Essentially, to prove that cardiomyopathy is due to mitochondrial genetic abnormalities, it is necessary to prove mitochondrial abnormalities in the myocardium by pathology or tissue biochemistry, but since an autopsy was not obtained in this case, we consider it a cardiac complication of systemic mitochondrial disease due to the pathogenic mutation of *PARS2*.

## Conclusion

In previous studies, most *PARS2* variants have been linked to neurological disorders (10, 27). Reports of patients demonstrating heart failure and DCM are rare. This is the first case to reveal a novel compound heterozygous variant of *PARS2*-induced lethal cardiomyopathy with unreversed heart failure. Thus, this report enhances our understanding of mitochondrial tRNA function in maintaining heart function. Furthermore, the case report extends the clinical manifestation spectrum of *PARS*-associated mitochondrial disorder, and early cardiac assessments and advanced myocardial therapy would benefit such patients.

## Data Availability

The datasets presented in this study can be found in online repositories. The names of the repository/repositories and accession number(s) can be found in the article/Supplementary Material.
